# Did Advances in Global Surveillance and Notification Systems Make a Difference in the 2009 H1N1 Pandemic?–A Retrospective Analysis

**DOI:** 10.1371/journal.pone.0059893

**Published:** 2013-04-03

**Authors:** Ying Zhang, Hugo Lopez-Gatell, Celia M. Alpuche-Aranda, Michael A. Stoto

**Affiliations:** 1 Department of Health Systems Administration, Georgetown University, Washington, District of Columbia, United States of America; 2 National Institute of Public Health, Cuernavaca, Mexico; The University of Hong Kong, China

## Abstract

**Background:**

The 2009 H1N1 outbreak provides an opportunity to identify strengths and weaknesses of disease surveillance and notification systems that have been implemented in the past decade.

**Methods:**

Drawing on a systematic review of the scientific literature, official documents, websites, and news reports, we constructed a timeline differentiating three kinds of events: (1) the emergence and spread of the pH1N1 virus, (2) local health officials’ awareness and understanding of the outbreak, and (3) notifications about the events and their implications. We then conducted a “critical event” analysis of the surveillance process to ascertain when health officials became aware of the epidemiologic facts of the unfolding pandemic and whether advances in surveillance notification systems hastened detection.

**Results:**

This analysis revealed three critical events. First, medical personnel identified pH1N1in California children because of an experimental surveillance program, leading to a novel viral strain being identified by CDC. Second, Mexican officials recognized that unconnected outbreaks represented a single phenomenon. Finally, the identification of a pH1N1 outbreak in a New York City high school was hastened by awareness of the emerging pandemic. Analysis of the timeline suggests that at best the global response could have been about one week earlier (which would not have stopped spread to other countries), and could have been much later.

**Conclusions:**

This analysis shows that investments in global surveillance and notification systems made an important difference in the 2009 H1N1 pandemic. In particular, enhanced laboratory capacity in the U.S. and Canada led to earlier detection and characterization of the 2009 H1N1. This includes enhanced capacity at the federal, state, and local levels in the U.S., as well as a trilateral agreement enabling collaboration among U.S., Canada, and Mexico. In addition, improved global notification systems contributed by helping health officials understand the relevance and importance of their own information.

## Introduction

In the past decade, many new advanced systems for disease surveillance and notification have been developed and implemented throughout the world [1]. These generally fall into two categories. *Indicator-based surveillance systems* gather and analyze original data, especially those indicative of emerging health problems in the population [2]. Recent advances include enhancements of traditional case reporting and laboratory capabilities, as well as the development and implementation of “syndromic surveillance” systems that collect and analyze statistical data on health trends – such as symptoms reported by people seeking care in emergency departments or other health care settings – or even sales of prescription or over the counter flu medicines or web searches [3]. *Notification systems*, on the other hand, provide a means for communicating about the evidence that emerges from indicator-based surveillance systems in order to better understand the implications of local results and to enable a global response if warranted. Notification systems in large part stem from the adoption and implementation of the International Health Regulations (IHR) and include efforts such as the Global Public Health Intelligence Network (GPHIN), ProMED Mail, HealthMap, Argus, and Veratect (described below), which search the Internet and other sources to identify disease outbreaks that might not have been apparent to health officials. These systems, also known as “event-based surveillance” [2], have the potential to detect outbreaks based on indirect evidence of illness not reported to local health officials.

The outbreak of a novel strain of A(H1N1) influenza virus, A/California/7/2009, now referred to as pH1N1, provides an opportunity to see how well these systems functioned in practice as an integrated public health surveillance system. The epidemiology of pH1N1 has been well described elsewhere [4]–[6], and adding to this understanding is not the goal of this paper. Rather, taking advantage of this opportunity, the primary objective is to identify the strengths and weaknesses of current global disease surveillance and notification systems in order to improve their performance in the future. Specifically, we ask whether and how advances in global surveillance and notification systems put in place in the last decade made a difference in the public health response to the 2009 H1N1 pandemic. We also identify the policy implications of the findings for future enhancements to global surveillance and notification systems, as well as for how preparedness should be assessed.

As a secondary objective, this analysis illustrates the use of “critical event analysis,” part of the toolkit for systematic quality improvement (QI), a perspective called for in the U.S. National Health Security Strategy [7]. Emphasizing processes (chains of events that produce specific outcomes) and systems of people and information, the QI approach refers to a range of specific practices including procedures and system changes based on their effects on measurable outcomes, reducing unnecessary variability in outcomes while preserving system differences that are critical to the specific environment, continuous improvement rather than onetime initiatives, and critical event/failure mode analysis. The NHSS Implementation Guide further calls for the development, refinement, and wide-spread implementation of QI tools. In particular, this includes “efforts to collect data on performance measures from real incidents … analyze performance data to identify gaps, [and] recommend and apply programs to mitigate those gaps” [8].

## Data and Methods

**Figure 1:**
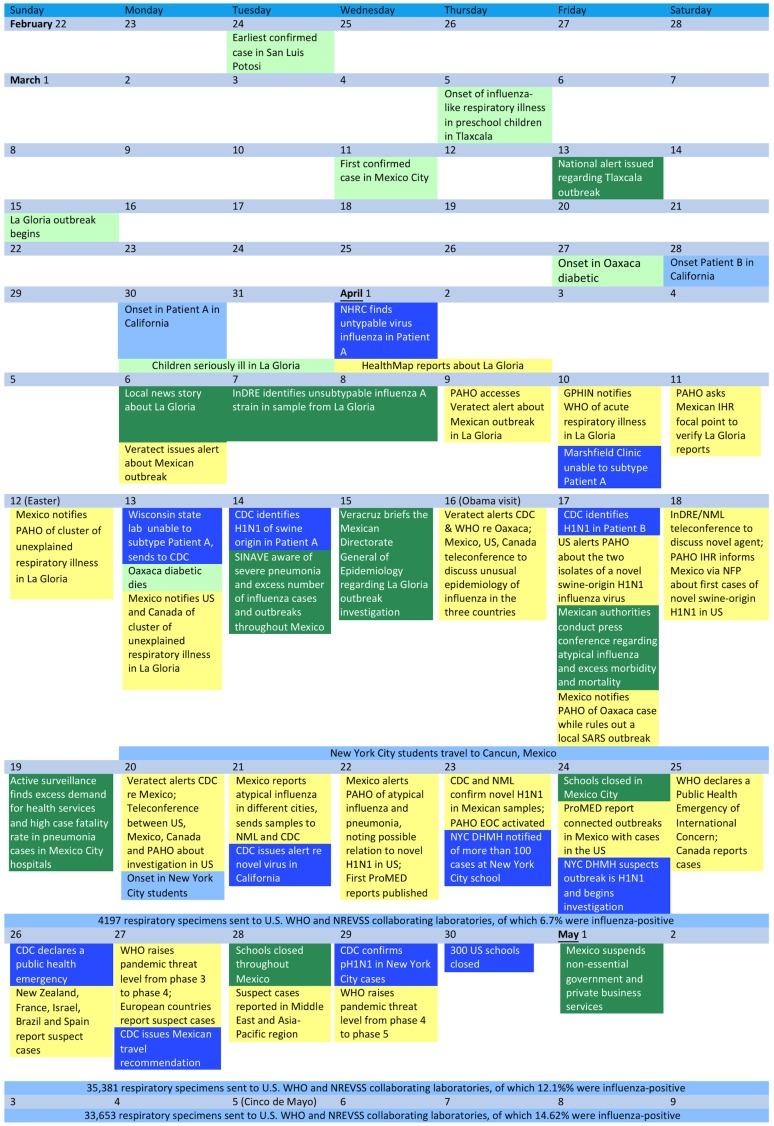
Timeline of H1N1 events. Note: Numbers in parentheses correspond to manuscript page where the event is described. Epidemiological events are indicated in light shades (green for Mexico and blue for the United States), local awareness and understanding of these events in dark shades (green for Mexico and blue for the United States), and global notifications and awareness of these events in yellow.

This analysis is an in-depth case study drawing on information from a systematic review of the scientific literature, official documents, websites, and news reports. In particular, we constructed a time line (Figure 1) in which three kinds of events are represented and distinguished by a color code (to be explained below): (1) the emergence and spread of pandemic H1N1 virus itself, (2) local health officials’ awareness and understanding of the emerging outbreak, and (3) notifications about and global health officials’ awareness of the events and their implications. The primary sources for this analysis were a timeline published by *Science*Insider, an on-line publication associated with *Science* magazine [9], other scientific and lay publications as indicated in the text, as well as two of the authors’ contemporaneous notes. In a number of cases the sources differed, so we used our judgment to see which fit best with the other time points. This uncertainty is represented in the text with phrases such as “In early April …”.

With the events classified in this way, we then conducted a “critical event analysis” focused on the surveillance process rather than the epidemiologic facts. Specifically, we first identified critical events, incidents that advanced the recognition of what we now know as a global pandemic. These events are points in time when the public health system might have responded sooner or later than it did, depending on the system’s capabilities. We then tried to identify the factors that allowed the events to occur when they did, rather than earlier or later, as in a root cause analysis. In particular, we asked (1) when health officials in Mexico, the United States, and at the global level became aware of the epidemiologic facts of the unfolding pandemic, (2) whether an earlier recognition could have been possible, (3) whether advances in surveillance notification systems seem likely to have hastened the detection of the outbreak, and (4) whether there are further improvements that might be possible through enhanced practices, procedures, or new systems. We sought to analyze decisions based on the information that was available, or could have been available, to the decision-makers at the time. Because illustrating the strengths and weaknesses of this approach is one of our objectives, we discuss the challenges, limitations, and opportunities presented by this approach in detail in the conclusions section.

## Results

### The Mexican Outbreak

The exact location of the first human cases of pH1N1 infection is not known, however retrospective analyses have identified cases dating back to February and March, 2009 in at least three locations throughout Mexico, *as indicated by the text in light green background in* *Figure 1*. The earliest confirmed cases occurred on February 24 in the state of San Luis Potosí in central Mexico [10] and the first confirmed case in Mexico City had its onset on March 11 [11]. There was also an outbreak of influenza-like illness in pre-school children in the State of Tlaxcala in central Mexico starting on March 5 [12]. Starting on March 15, a major respiratory disease outbreak occurred in La Gloria in the state of Veracruz. This outbreak was originally attributed to a large pig farm on the outskirts of town, but when three children became seriously ill in late March and early April, health authorities in Veracruz began to suspect an atypical influenza [9], [13]. Consistent with PAHO/WHO recommendations at the time, surveillance was conducted using use immunofluorescence (IFI), which has low sensitivity in practice. In addition, Mexico’s Institute for Epidemiologic Diagnosis and Reference (InDRE) used real-time polymerase chain reaction (rT-qPCR) for molecular diagnosis, but of course probes for the pandemic strain were not available until afterwards, so pandemic was not recognized at this time.

On March 27 a 39-year-old woman with newly onset diabetes mellitus in Oaxaca developed severe respiratory illness and eventually died of this illness on April 13 [14]. In addition, an excess amount of influenza-like illness was experienced in the Distrito Federal (Mexico City) in mid-March [9]. By mid-April, Mexican national health authorities were aware of these and other respiratory illness outbreaks throughout the country through the National Surveillance System SINAVE (Sistema Nacional de Vigilancia Epidemiologica). This system receives weekly reports on 117 notifiable conditions from nearly all of the more than 19,000 hospitals, clinics and doctors’ offices in Mexico and also monitors 520 sentinel influenza surveillance units covering all 32 states [15].

On March 13, the Mexican Directorate General of Epidemiology had issued an alert about the outbreak of influenza-like illness in pre-school children in the previous week in Tlaxcala [12](*the Mexican public health system’s awareness of the outbreak and response is represented by dark green background in* *Figure 1*). On April 6, a local news story reported that 60% of La Gloria residents were infected, with three deaths [13]. The following day, the InDRE identified an influenza A viral strain that was unsubtypable (i.e. a different strain than those known to be circulating at that time) in a sample from La Gloria. InDRE had previously identified unsubtypable samples from Mexico City, San Luis Potosí, and Baja California. By April 14, SINAVE was aware that there had been an increase in the number of cases and outbreaks of seasonal influenza observed since February [12], [16]. SINAVE was also notified through both official and unofficial channels of cases of severe laboratory-confirmed pneumonia, with high fatality, in young previously healthy adults between the ages of 20 and 40 years in Mexico City and the States of México, Veracruz and San Luis Potosí [9], [17]–[19]. Active surveillance of Mexico City hospitals starting on April 17 triggered by these reports found excess demand for health services and high case fatality rate in pneumonia cases [12].

The clinical and epidemiological characteristics of the cases that had come to light by mid-April varied, and respiratory illness during the winter could easily have been regarded as seasonal influenza. Many of the cases were determined to have influenza B, a trend that was also being observed in the United States [17]. But a severe respiratory infection that, unlike seasonal influenza, affected children and young adults together with prompts from the World Health Organization (WHO) as discussed below, led the Directorate General of Epidemiology to “connect the dots” between the outbreaks across the country by mid-April.

On April 15, Veracruz officials briefed the Directorate General of Epidemiology regarding La Gloria outbreak investigation, with Pan American Health Organization (PAHO) officers in attendance. Two days later, authorities conducted a press conference to warn about atypical influenza season with increasing morbidity trend and excess mortality. Following notification about the emergence of novel H1N1 in the United States and interactions with Canadian health officials and the U.S. Centers for Disease Control and Prevention (CDC) as described below, Mexico notified PAHO (the WHO regional office for the Americas) of a potential atypical pneumonia outbreak on April 22, closed schools in Mexico City on April 24 and throughout Mexico on April 28, and did not open high schools and universities until May 7. On May 1, non-essential government and private sector business services were suspended. The number of confirmed cases peaked shortly afterwards, but rebounded for a second peak in June and July, by which time the entire country was affected [20].

### The United States Outbreak

In late March, a 9-year old girl and a 10-year old boy in southern California became ill with influenza (*U.S. epidemiological dates are represented by text with light blue background in* *Figure 1*) [21]. An experimental diagnostic device was being tested by the Naval Health Research Center (NHRC) in San Diego requiring that respiratory samples be collected and analyzed. On April 1, NHRC found an unsubtypable influenza A virus in one of these samples (*the U.S. public health system’s awareness of the outbreak and response is represented by text in dark blue background in* *Figure 1*). By protocol, respiratory samples were sent to the designated reference laboratory, the Marshfield Clinic in Wisconsin, which on April 10 confirmed that the pathogen was influenza A virus, but could not identify the strain any further. Also following protocol, a part of the sample was sent to the Wisconsin State Laboratory of Hygiene, which confirmed the finding on April 13 and forwarded the sample to CDC for further analysis. On April 14, CDC identified the subtype as H1N1 of swine origin, and on April 17 found swine influenza A (H1N1) virus in another specimen from Naval Health Research Center in San Diego. Following a call with California health officials on April 19, CDC issued an alert and notified WHO on April 21 [9].

Between April 10 and 19, 14 students from a high school in Queens, New York travelled to Mexico (all but one to Cancun) during their Spring recess, and developed flu symptoms in the week of April 19. On April 23, two days after the CDC alert, the school nurse notified the New York City Department of Health and Mental Hygiene (DHMH) that approximately 100 students were being sent home with flu symptoms. DHMH notified CDC that afternoon and began an investigation on April 24. The following day, DHMH reported that most laboratory specimens from these students tested positive by rt-PCR for influenza A with no human H1 or H3 subtypes detected, indicating that the virus was probably pH1N1. On April 29 CDC confirmed by rt-PCR that most specimens were positive for pH1N1 [22].

On April 26, aware of the New York outbreak, as well as 20 cases from California and Texas, U.S. Department of Health and Human Services (HHS) declared a public health emergency in the United States [23], and on the following day, CDC issued “a travel health warning recommending that United States travelers postpone all non-essential travel to Mexico” [24]. About three hundred schools in the US were closed by April 30 when the accumulated pH1N1 cases were over 100 nationwide. An immediate consequence was an increase in the number of U.S. respiratory specimens sent for testing by the WHO and NREVSS collaborating laboratories from 4219, of which 7.7% were influenza-positive, in the week ending April 25 (week 16) to 14,330, of which 13.2% were influenza-positive, in the week ending May 2 (week 17). The number of specimens, along with the percent influenza-positive, peaked at 7844 and 41.9%, in the week ending June 20 (week 24). By the end of the summer the first wave had waned, but pH1N1 cases had been confirmed in every U.S. continental state [25]–[27].

### International Awareness and Global Spread

Health officials outside of Mexico were potentially aware of what was eventually determined to be the 2009 H1N1 pandemic as early as April 1, when HealthMap first disseminated local media reports about a “mysterious” influenza-like illness in La Gloria (*global epidemiology and response represented in text with yellow shading in* *Figure 1*). The HealthMap system combines automated, around-the-clock data collection and processing with expert review and analysis to aggregate reports according to type of disease and geographic location. HealthMap sifts through large volumes of information on events, obtained from a broad range of online sources in multiple languages, to provide a comprehensive view of ongoing global disease activity through a publicly available Web site [28]. Throughout the month of April, HealthMap also identified informal local Spanish-language sources reporting on the spread of the epidemic though Mexico.

On April 6, Veratect, a private firm based in Kirkland, Washington that conducts disease surveillance, issued an alert based on information from La Gloria and other sources of "strange" outbreak of acute respiratory infection, which led to bronchial pneumonia in some pediatric cases. This alert was available to CDC, WHO, PAHO and several US city and state public health officials that subscribe to Veratect’s service, and records indicate that PAHO accessed it on April 9 and 10 (Wilson, personal communication, December 3, 2009).

On April 10, GPHIN notified WHO of acute respiratory illness in La Gloria [28], and on the following day the PAHO IHR focal point (the point of contact with the WHO under the IHR) requested verification. On Sunday, April 12, Mexico's director general of epidemiology, Hugo López-Gatell, who served as the Mexican IHR focal point, confirmed the existence of acute respiratory infections, but said the initial epidemiological investigation produced no evidence of a link to fecal contamination of pig farms [9]. Dr. López-Gatell considered this outbreak to be a potential “public health emergency of international concern" (PHEIC) because it met IHR criteria (severe public health impact and an unusual event) and provided a detailed report to PAHO. On April 13, based on a tri-lateral collaboration agreement, this communication was shared with the IHR focal points for the U.S. and Canada, and was discussed in a teleconference on April 16. Concerned that this pattern was similar to SARS, WHO requested verification [29], and Mexican authorities quickly responded that “lab tests had failed to find any connection to a SARS-like or even a flu virus.” On April 17, Dr. López-Gatell sought information from local officials about a cluster of cases of acute respiratory illness in a hospital in Oaxaca and was told that there was no cluster, but rather a single patient with diabetes with a severe case of acute respiratory illness, presumably of viral origin [17]. The same day, Mexico notified PAHO of this case, noting the possibility that it could be related to the cases of novel H1N1 in the United States.

On April 17, InDRE director Celia Alpuche contacted the Canadian National Microbiology Laboratory (NML), part of the Public Health Agency of Canada (PHAC), for help in dealing with the situation that was developing. Dr. Alpuche knew NML’s director, Frank Plummer, through the Global Health Security Action Group and other international collaborations, and valued his expertise dealing with SARS and other unknown pathogens. In a teleconference the following day InDRE and NLM officials concluded that the outbreak was likely to be a novel agent, unrelated to influenza. Following a conference call between Mexico, Canada, the United States, and PAHO, Mexican samples were sent to NML and CDC, and on April 23, both labs identified the viral subtype as the novel H1N1. This collaboration was possible because of the Security and Prosperity Partnership of North America (SPP), a trilateral agreement between the United States, Canada, and Mexico launched in March 2005. The CDC MMWR report on two California children confirmed with Swine influenza A were posted on the ProMED website on April 21, which was the first report regarding the novel H1N1 influenza virus [30]. On April 24, ProMED also reported severe respiratory illness clusters in Mexico and connected it with the U.S. cases [31].

Aware of the developments in Mexico and Canada, Veratect attempted to contact CDC, California, and Texas officials on April 16 and 17. On April 20, Veratect urgently attempted to contact CDC. James Wilson, Veratect’s medical director, said in December, 2009 that he had been more concerned about this situation than any other in many years of surveillance work (Wilson, personal communication, December 3. 2009). However Dr. Wilson was quoted in the *Washington Post* on May 3, 2009 as having said “I suspect this is probably a false alarm.”

On April 22, Mexico’s IHR focal point alerted PAHO about an unusual outbreak of atypical pneumonia in young adults and indicated a probable relation of these events to the outbreak in La Gloria [32]. On April 25, the Mexican epidemiological evidence, together with the laboratory results confirming the pH1N1 subtype in both Mexican and U.S. cases, led the WHO to declare a “Public Health Emergency of International Concern” [33]. Over the next few days, Canada, plus a number of countries in Europe, the Middle East, and the Asia-Pacific regions reported suspected cases. Reflecting the rapid spread of the virus, the WHO raised the global pandemic threat level from phase 3 to phase 4 on April 27. By May 6, WHO had reported 1,893 confirmed cases in 23 countries. Since cases were identified in these countries so shortly after reagents were available for testing, it is likely that the virus was actually circulating days to weeks earlier.

## Discussion

A/California/7/2009, now known as pH1N1, was circulating in Mexico and the United States in March 2009 and perhaps earlier. That it was a novel pathogen came to the world’s attention in April because of three critical events: the recognition that multiple apparently disparate disease outbreaks throughout Mexico were connected, the identification of novel pH1N1 in two California children and its subsequent connection to the Mexican cases, and the recognition that an outbreak in New York City was connected to the Mexican and California cases.

The first critical event was the identification of pH1N1 in two California children through the NHRC’s surveillance research program. Because the epidemiologic information suggested human-to-human transmission, this triggered a series of events involving three laboratories (the Marshfield Clinic, the Wisconsin State Laboratory of Hygiene, and eventually the CDC, which identified the pathogen). Although the first child became ill on March 28, CDC did not identify pH1N1, a potential public health emergency of international importance under the IHR, until the second child was determined to also have pH1N1 on April 17, three weeks later and five days after Mexico had notified a potential PHEIC regarding the La Gloria outbreak. In retrospect, one might ask whether this identification could have occurred earlier. A review of the timing of the events suggests that it could have, but only if health officials in California, Wisconsin, and the CDC knew it was a novel pathogen, which of course they did not know. To find two children with unsubtypable influenza at the end of the flu season is not remarkable, and indeed it is only because of the research being conducted at NHRC that these cases came to light at all.

The second critical event (which actually started earlier than the first) was the recognition that a number of disease outbreaks throughout Mexico with apparently different epidemiological characteristics represented a single phenomenon and thus were a potential public health event of international concern. Health authorities in Veracruz and Tlaxcala were aware of outbreaks with an unusual high frequency of severe pneumonia in otherwise healthy young people in March, and in the week of April 5 national authorities came to realize that the outbreaks were related, resulting in the first international alert on Sunday, April 12. However it was not recognized that the responsible pathogen was pH1N1 until April 23, two days after CDC identified the new virus strain and published its alert about pH1N1 in the California children. Two labs in Canada and the United States were able to test samples from Mexico and determine that pH1N1 was the pathogen quickly, in only two days.

Although the samples were sent earlier than the established protocol in response to Mexican authorities growing concerns, one might ask whether samples could or should have been sent for testing earlier. As indicated in Figure 1, during the week of April 12, which happened to begin on Easter Sunday coincidentally included a visit of President Obama to Mexico City, CDC was identifying pH1N1 in the first two cases and Mexican authorities were conferring with PAHO and their North American counterparts about the situation. Although GPHIN, HealthMap, Veratect, and GPHIN had been issuing alerts about events in Mexico for more than a week, no one seems to have connected the outbreaks in Mexico and the United States until early in the week of April 19. Had that connection been made earlier it is possible that WHO could have declared a “Public Health Emergency of International Concern” before Saturday April 25. Mexican, U.S., Canadian officials held a trilateral teleconference on April 16, but U.S. participants did not mention the isolation of novel pH1N1 about which they alerted PAHO one day later. Given the uncertainties and the concern that both Mexican and American health officials must have had about the situation in their own countries during the week of April 12, it is understandable that they did not make the connection. Only in retrospect did it become clear that each had the key to the other’s epidemiologic puzzle.

The final critical event was the recognition, on April 24, of an outbreak of pH1N1 in New York City high school students who had travelled to Cancun, Mexico during their Easter recess the previous week. This recognition, only days after the first student became ill, was possible because a school nurse and New York City health officials were aware of pH1N1 and the Mexican situation through alerts and the news media earlier in the week of April 19. Although the New York Department of Health and Mental Hygiene would have definitely investigated an event of this magnitude, knowledge of the CDC and Mexican alerts a few days earlier added urgency to the situation (Fine, personal communication, Feb. 5, 2011). This in turn contributed to understanding the outbreak’s epidemiology and presumably helped trigger the declaration of a health emergency in the United States on April 26 as well as the WHO’s alert the previous day. Since the report was filed immediately after the students became ill, and immediately acted upon, it seems unlikely that this could have happened any earlier.

In retrospect, considering the chain of critical events, if the California samples been tested with more urgency in the week of April 5 rather than April 12 and the results reported to Mexican authorities earlier, it seems possible that global alerts about pH1N1 could have been advanced by about one week to April 18. By this time, however, the virus had spread throughout Mexico and the United States, especially because of Easter travel. So even with the earliest possible recognition of the emerging pandemic, it seems unlikely that world-wide spread could have been contained. And of course what now seems clear in retrospect was far from clear in April, 2009. Indeed, coming at the end of the normal flu season, no single Mexican or American surveillance finding was exceptional, so without the international communication that occurred in 2009 the pandemic could have taken longer to detect and to characterize than it did.

Although it is impossible to quantify the effect, it could have taken much longer for the world to become aware that a new pandemic subtype had emerged. One must only consider the years of effort it took to identify and characterize HIV three decades ago, and the resulting confusion [34]. Global recognition of the emergence of SARS in 2003 five years earlier was delayed for weeks despite some awareness of its effect in China [35]. In their analysis of 281 WHO-verified non-endemic human infectious disease outbreaks that occurred between 1996 and 2009, Chan and colleagues found that the median time from outbreak start to outbreak discovery decreased from 29.5 days in 1996 to 13.5 days in 2009, and the median time from outbreak start to public communication about the outbreak decreased from 40 days in 1996 to 19 days in 2009 [36]. Both the Mexican and the U.S. responses compare favorably to these statistics, and our analysis of the impact of notification systems is consistent with Chan and colleagues’ hypothesis that the improvement was largely due to the proliferation of Internet-based notification systems. Chan and colleagues’ analysis, however, only addresses the recognition of single outbreaks. Recognizing that the same pathogen was responsible for outbreaks at various locations throughout Mexico and in Southern California and New York City, and moreover that the pathogen was a newly emerged viral subtype, is more challenging. It is rare for subtypes to be identified so quickly (Morens, personal communication, February 5, 2011), but modifications in the protocol to assess the importance of non-subtypable strains before its definitive confirmation may provide opportunities for more timely responses.

Analysis of these critical events shows how global investments in disease surveillance and notification, coupled with a heightened awareness of pandemic influenza, contributed to an enhanced public health response to pH1N1. First, enhanced laboratory capacity in the United States and Canada led to earlier identification and characterization of the novel H1N1 strain. Among other things, this recognition triggered national and global pandemic plans, PCR-based tests were quickly developed to aid in surveillance and clinical decision-making, and a vaccine seed strain was quickly developed that led to the development of pandemic vaccine in time to be used during the second pandemic wave in the Fall of 2009 (although not before that wave began), in which the CDC had been taking a leading role. In particular, the early detection was due in large part to the existence of an experimental influenza surveillance system developed and operated by the U.S. Navy’s NHRC in Southern California, which identified the first two cases. Laboratory response networks initiated or enhanced in recent years were also critical because they enabled the involvement of CDC and Canada’s NLM, which had the capacity to recognize pH1N1 as novel. This includes the collaboration among Mexico’s InDRE, the NLM, and the CDC that was possible because of the Security and Prosperity Partnership of North America (SPP) agreement as well as protocols and relationships that facilitated collaboration among the NHRC, the Marshfield Clinic, the Wisconsin State Laboratory of Hygiene, the New York City Department of Health and Mental Hygiene, and the CDC.

Second, enhanced global notification systems led to earlier detection and characterization of the outbreak by helping to “connect the dots between cases in California, Mexico, and New York City.” Through SINAVE and other sources, Mexican officials were aware, for instance, of a serious problem in the week of April 12, but it was not until CDC’s publication regarding pH1N1 in California the following week that they sent samples and realized that the two outbreaks were the same. After the pandemic and with the support of the US and Canada, Mexico has also developed its own capabilities for rt-PCR testing throughout the country, facilitating much faster diagnosis. Similarly, without the awareness that the same virus that was making children ill in California and circulating widely – and seriously affecting young people – in Mexico, during the week of April 19, the school nurse in Queens and New York City health officials might not have taken the outbreak in students who had travelled to Mexico the previous week as seriously. The notification systems that contributed to these results include the International Health Regulations, voluntary reporting systems such as ProMED, as well as active searching activities GPHIN, HealthMap, Argus, and Veratect. In addition, some have speculated that countries’ awareness that outbreaks within their borders will soon come to light through these channels, increases the likelihood that they will report themselves [37].

The early events in the 2009 H1N1 pandemic thus illustrate the important contribution of the 2005 IHR and the paradigm shift that accompanied it. This includes, the definition of a PHEIC as a comprehensive and flexible representation of health hazards, the algorithm for risk assessment (Annex 2 of the IHR), and the existence of National Focal Points that can (and are mandated to) communicate directly with the WHO rather than go through diplomatic channels. In this experience, the IHR system was also instrumental to speeding two-way communications between Mexico and PAHO and between the US and PAHO. Similarly, the North American Plan for Avian and Pandemic Influenza (NAPAPI) facilitated communication among Mexican, U.S., and Canadian health authorities. On the other hand, Mexico, Canada, and the U.S. currently have no official protocols for sharing information from event-based surveillance sources such as GPHIN, HealthMap and Veratect. The second edition of the North American Plan for Animal (formerly Avian) and Pandemic Influenza (NAPAPI), published in April 2012, seeks to develop a more effective international sharing mechanism based on the lessons from the 2009 pandemic.

Syndromic surveillance systems played an important role in detecting the pH1N1outbreak, but a different the role that is commonly used to justify them – that such systems can detect outbreaks before conventional surveillance systems and enable a rapid public health response [3]. Because pH1N1 emerged during the normal flu season, there were too few cases to have been detected by standard alerting algorithms. In the U.S., for instance, the earliest appearance of the pandemic did not trigger a quantitative alert in any syndromic surveillance system, although four of the earliest cases presented at providers who were members of CDC’s ILINet and so were tested and flagged for attention [38]. In Mexico, however, general acute respiratory illness with no lab diagnosis is a notifiable condition. A sharp increase in such reports to SINAVE in early April, along with an analysis indicating an atypical age-distribution [19], helped Mexican officials realize that the problem they were seeing was widespread, and led authorities to conduct active surveillance for severe pneumonia starting on April 17 and eventually influenza-like illness (ILI) in patients visiting primary healthcare units and hospitals as well as influenza-related deaths [6].

### Conclusions

An analysis of this sort is clearly limited in two important respects. First, since public health experts in the midst of puzzling out the facts of a disease outbreak rarely take notes – indeed it is often not clear until days or weeks into an outbreak that there is anything worth recording – any retrospective analysis is subject to recall bias colored by the epidemiological data and explanations that eventually emerged [39]. For instance, facts and events that might not have seemed important in isolation at the time take on added significance after the fact if they fit the epidemiological story that was eventually constructed. Second, it is impossible to know what would have occurred in counter-factual circumstances – if, for example, a certain surveillance system had not existed. For instance, the fact that by 2009 the world was four to five years into a period of enhanced concern about pandemic influenza means that even in the absence of any concrete surveillance and notification enhancements, it is likely that the public health response was better than what might have been expected before the avian influenza outbreak that started in Hong Kong in 1997 and the SARS outbreak in 2003.

Despite these limitations, a systematic analysis of three critical events that occurred during March and April 2009– identification of pH1N1 in samples from two children from California, the recognition that multiple apparently disparate disease outbreaks throughout Mexico represented a single phenomenon related to the California cases, and the recognition that an outbreak of influenza in New York City high school students were part of the same picture – shows that both enhanced laboratory-based surveillance, coupled with improved global notification systems, did seem to have improved the global public health response to pH1N1. The surveillance enhancements that made this possible include an experimental influenza surveillance system operated by the NHRC in Southern California as well as laboratory response networks linking Mexico’s InDRE, Canada’s NLM, and the CDC, as well as private and public health laboratories in the United States. The global notification systems that contributed to these results include formal and informal channels as well as activities such as GPHIN, HealthMap, ProMED Mail, Argus, and Veratect, which actively search the Internet for evidence of disease outbreaks. At the national level, starting in May, 2008, Mexican authorities held a weekly meeting, named “Epidemiologic Pulse,” to scan and assess epidemiological events in Mexico and the world. This session played a key role in integrating the information from formal and informal sources that emerged nearly a year later. PAHO officials attended the April 15 session at which the La Gloria situation was discussed. The trilateral teleconference the following day was enabled by the North American Plan for Avian and Pandemic Influenza (NAPAPI), a non-legally-binding agreement prepared under the Security and Prosperity Partnership of North America treaty. Since most of this did not exist a decade earlier, is seems likely that the investments in building these systems, together with a heightened awareness of pandemic influenza, enabled a more rapid and effective global public health response to H1N1.

Considering the chain of critical events, it is possible that global alerts about pH1N1 could have been advanced by about one week to April 18. But since the virus had already spread throughout Mexico and the United States and elsewhere by this time, it seems unlikely that this would have made a difference in containing the world-wide spread of the virus. Rather, recognizing that there are many false positives in epidemiology, and what now seems clear in retrospect was far from clear in April, 2009, the picture that emerges from this analysis is a global public health system, and particularly public health agencies in Mexico, Canada, and the United States, that worked together effectively to solve a challenging epidemiologic puzzle in a reasonably timely fashion.

This analysis also illustrates the challenges of early detection and characterization in public health emergencies. First, although in retrospect the events described in this analysis clearly add up to tell the story of the emergence of a new pandemic viral subtype, many of the events – even large numbers of respiratory illness cases at the end of the winter flu season – taken in isolation were not sufficient to cause alarm. Given the number of such “signals” that truly are isolated events, it is not useful or appropriate for local, national, or international public health agencies to react with alarm on every such occasion. Second, as with most novel pathogens, the emergence of pH1N1 was characterized by uncertainty that took weeks to months to resolve. Many emergency preparedness professionals, however, still think in terms of single cases triggering a response in hours or at most days and this thinking is reflected in such key public health preparedness documents as CDC’s 2011 *Public Health Preparedness Capabilities: National Standards for State and Local Planning* [40].

Epidemiologists familiar with the emergence of novel pathogens rightly compare the rapidly evolving facts and scientific knowledge to the “fog of war,” [41], and the United Kingdom’s Pandemic Influenza Preparedness Programme has shown how it should be factored into public health preparedness planning [42]. Similarly, recognition that it may take time to understand and characterize an emerging threat has important implications for implementation of the International Health Regulations, which define a “public health event of international importance” (PHIEC) through a flow chart [37], [43] that implicitly presumes a bright line between a PHIEC and other outbreaks.

More broadly, this recognition means that it is important to expect and plan for uncertainty in preparing for the emergence of a new pathogen. This requires attention to response *capabilities* in addition to preparedness *capacities*. For instance, CDC’s and the Trust for America’s Health’s most recent state-by-state assessments of public health preparedness focus on ensuring that state and local public health laboratories can respond rapidly, identify or rule out particular known biological agents, and have the workforce and surge capacity to process large numbers of samples during an emergency [44], [45]. Although such capabilities seem necessary for some events they are not sufficient, and none of these measures would have ensured that the public health system could have identified the emergence of and characterized pH1N1 as well and as efficiently as it was done in Mexico and the United States in April 2009. Rather, the surveillance system capabilities that were most essential were the availability of laboratory networks capable of identifying a novel pathogen, notification systems that made health officials aware of the epidemiological facts emerging from numerous locations in at least two countries, and the intelligence necessary to “connect the dots” and understand their implications.

Finally, this analysis illustrates the potential of the critical events approach for collecting, analyzing, and understanding the policy implications of data from real incidents on public health system’ emergency response capabilities. There are three critical components of this approach; each requires knowledge of public health systems and professional judgment.

First, the analyst must prepare a timeline describing key events in both the epidemiology and the public health response, such as the one in Figure 1. This can be a challenge because, as noted above, early events are not in and of themselves seen as noteworthy and are typically not recorded as they occur. Situation reports that are now routinely prepared by emergency response organizations can be helpful, but are typically not started until there is an indication of a problem. For instance CDC did not activate its Emergency Operations Center for pH1N1 until April 22, 2009, which was more than a month after the outbreak began in Mexico. Alternatively, it would be useful to retrospectively record the officials’ knowledge and understanding of events as soon as possible after it becomes clear that a public health emergency is underway.

Second, based on this timeline, one must identify the critical events. These are events of more complexity than the recognition of a cluster of cases, but less than the emergence of a new pathogen. They represent opportunities when the response might have occurred sooner or later than it did, depending on the public health system’s capabilities. This is comparable, in a standard root cause analysis (RCA) to the choke points in the process map when errors occur. For this analysis, for instance, we choose incidents that advanced the recognition of, and enabled a response to, the global pandemic. Identifying these events was challenging, but the creation and careful of a timeline was an essential first step.

Finally, the analyst must identify the factors that allowed the events to occur when they did, rather than earlier or later. In a standard RCA, these are the “root causes.” This requires knowledge of how public health systems are supposed to perform and the factors that can degrade this performance. It is also useful to consider what might have happened had the critical events turned out differently, an approach that March and colleagues describe as “simulating experience” [46].

Learning about public health systems’ emergency response capabilities is challenging because actual events are unique, and both the epidemiological facts and the context varies from one community to another. In other words, there is no replication, a centerpiece of the scientific method. In this context, our analysis of the global public health system’s ability to detect the pH1N1 pandemic gains rigor not by statistical analysis of repeated events but rather by a detailed analysis of the timing of events in Mexico, the United States, and the rest of the world. The kind of analysis described here is far more extensive and probing than is commonly seen in the After Action Reports (AARs) prepared by health departments after exercises or actual events [47], and illustrates the potential of the critical events approach for learning about public health system’ emergency response capabilities from real incidents that the NHSS Implementation Guide calls for [8].
